# Age differences in the impact of forced swimming test on serotonin transporter levels in lateral septum and dorsal raphe

**DOI:** 10.1186/1744-9081-10-3

**Published:** 2014-02-03

**Authors:** Rosa-Elena Ulloa, Aliyeri Díaz-Valderrama, Jaime Herrera-Pérez, Martha León-Olea, Lucía Martínez-Mota

**Affiliations:** 1 Instituto Nacional de Psiquiatría Ramón de la Fuente Muñiz, Calzada México-Xochimilco 101, Col. San Lorenzo Huipulco, Delegación Tlalpan, Mexico City 14370, Mexico; 2 Hospital Psiquiátrico Infantil “Dr. Juan N Navarro”, San Buenaventura 86, Col. Belisario Domínguez, Delegación Tlalpan, Mexico City 14080, Mexico

**Keywords:** Despair, Forced swimming test, Rats, Serotonin transporter, Age differences

## Abstract

**Background:**

Forced swimming test (FST) is an animal model which evaluates behavioral despair and the effect of antidepressants such as the selective serotonin reuptake inhibitors; the FST modifies the expression of some receptors related to antidepressant response, but it is not known whether serotonin transporter (SERT), their main target, is affected by this test in animals of different ages. Antidepressant response has shown age-dependent variations which could be associated with SERT expression. The aim of the present study was to analyze changes in the SERT immunoreactivity (SERT-IR) in dorsal raphe and lateral septum of male rats from different age groups with or without behavioral despair induced by their exposure to the FST, since these two structures are related to the expression of this behavior.

**Methods:**

Prepubertal (24 PN), pubertal (40 PN), young adult (3–5 months) and middle-aged (12 months) male rats were assigned to a control group (non-FST) or depressed group (FST, two sessions separated by 24 h). Changes in SERT-IR in dorsal raphe and lateral septum were determined with immunofluorescence.

**Results:**

Pubertal and middle-aged rats showed higher levels of immobility behavior compared to prepubertal rats on the FST. SERT-IR showed an age-dependent increase followed by a moderate decrease in middle-aged rats in both structures; a decreased in SERT-IR in lateral septum and dorsal raphe of pubertal rats was observed after the FST.

**Conclusions:**

Age differences were observed in the SERT-IR of structures related to behavioral despair; SERT expression was modified by the FST in lateral septum and dorsal raphe of pubertal rats.

## Background

Serotonin transporter (SERT) is responsible for terminating the serotonin (5-HT) action in the extracellular space by its reuptake into presynaptic terminal, controlling the availability of this neurotransmitter in the synaptic cleft [1]. This protein is the main target of the selective serotonin reuptake inhibitors (SSRIs), which bind SERT blocking its activity; this action allows an increase in 5-HT levels in the synaptic cleft and in serotonergic neurotransmission, being this first step for the antidepressants’ long-term effect [2,3]. Several authors proposed that SERTs are the primary regulators of the serotonergic transmission and that the effect of SSRIs may be related with their number [4].

Serotonin regulation shows age-dependent adaptations; 5-HT uptake measured in animal studies is higher in the developing brain as compared with adult values [5]. In rats mRNA for SERT can be determined by embryonic day 13, and the uptake of 5-HT reaches adult levels at birth in brain synaptosomes, at five weeks of age the amount of uptake is doubled and then decreases to adult levels again [6]. Specific areas, such as the median raphe exhibit a 25% decrease of SERT density in adults compared to prepubertal rats [7]. SERT binding in 3 to 18 years old children and adolescents shows an increase [8], followed by a decrease at the approximate rate of 10 percent per decade [9].

SERT can be modified by stress, where a reduction of mRNA in the raphe pontis was observed [10]. FST is an extensively used model in which a behavioral change is induced by acute stress: After a pretest 15-min session, rodents show an increased immobility 24 h later in the 5-min test. The increased immobility reflects despair, a depressive-like behavior [11], which is reduced by antidepressant drugs [12,13]. A 5-min session significantly increases the 5-HT output in the median raphe nuclei [14]; more recently, it was demonstrated that the FST increases membrane potential excitability and regulates the modulation of glutamatergic afferents on dorsal raphe neurons, these changes could alter their ability to process incoming signals and distribute them to their distinct forebrain targets [15]. Dorsal dorsal raphe sends projections to lateral septum [16]. In FST, the discharge rate of serotoninergic neurons in dorsal raphe was attenuated by the CRH of local GABAergic neurons [17]. In lateral septum, a 5-HT decrease after pretest followed by an increase after the test session have been related with despair [18], thus immobility was positively correlated and swimming was negatively correlated with changes in extracellular 5-HT in this structure [19]. In addition, an attenuated and enhanced firing rate in lateral septum was related with despair and the response to antidepressants, respectively [20].

Numerous evaluations of fluoxetine (an SSRI) have revealed an optimal antidepressant response in young adult male rats which is not observed in other ages, i.e., prepubertal rats show no antidepressant-like response in the forced swimming test (FST) while aged rats exhibit an attenuated antidepressant-like effect [21,22]. These variations may be explained by age-dependent changes on SERT expression which could account for the variations on its susceptibility to be affected by stress.

With this basis, the aim of the present study was to analyze changes in the SERT immunoreactivity (SERT-IR) in dorsal raphe and lateral septum of control or FST submitted male rats from different age groups.

## Methods

### Animals

Male Wistar rats from the vivarium of the Instituto Nacional de Psiquiatría Ramón de la Fuente Muñiz were housed 4–8 per cage in polycarbonate boxes according to age on an inverted 12-h light/dark schedule in a temperature-controlled (22°C) room. All animals had ad-libitum access to food and water. The rats were classified by age in prepubertal (24–32 PN, weight 90 g, n = 7), pubertal (40–41 PN, considering preputial separation to distinguish the onset of pubertal age; weight 120 g, n = 8), young adult (3–5 months; weight 370 g, n = 9) and middle age (12–14 months; weight 600 g, n = 8), considering as a reference the reproductive status in these ages [23]. All experimental procedures were performed in accordance to general principles of laboratory animal care [24] and the Mexican official norm for animal care and handling (NOM-62-ZOO-1999) [25]. The experimental protocol with laboratory animals was elaborated taking into account the 3R principles, and was approved by the ethical committee of Instituto Nacional de Psiquiatría Ramón de la Fuente Muñiz (NC093370.1).

### Experimental design

Animals were randomly assigned to FST or control group (non-FST groups, 4 subjects per group). Thirty minutes after the second session of FST, rats were anesthetized and perfused and their brains were removed and preserved. The control animals remained in the same housing and care conditions and perfused at the same time than the FST group.

### Forced swimming test

For this study the modified version of the FST was used [13]. Swimming sessions were conducted by placing rats in individual glass cylinders (46 cm height × 20 cm diameter) containing water at 23-25°C, 30 cm deep. Groups assigned to FST were subjected to the 15 min pre-test followed by a 5 min test 24 h later, which was videotaped. The sessions were run between 1200 and 1400 h. A time-sampling technique was used to score, every 5 s, the presence of immobility (floating without struggling and making only those movements necessary to keep the head above the water), swimming, active motions (moving and diving around the jar) or climbing (active movements with forepaws in and out the water, usually directed against the wall). Results were expressed as mean number of counts ± s.e.m. of the behaviors each 5 min. Inter- and intra-rater reliability was at least r = 0.87 for scoring FST behaviors by two observers.

### Open field test (OFT)

An ambulation test was conducted in order to discard an influence of locomotor activity on the results of the FST. Independent groups of prepubertal, pubertal, young adults, and middle aged male rats (n = 10 per group) were evaluated in an automatic system (PanLab) consisting of a Plexiglass cage (45×35×45 cm) with two infrared sensors located on the cage walls (2.5 and 10.5 cm from the cage base), coupled to IR LE8811 software. The system detected all rats’ ambulatory movements and registered the movement numbers (counts) in a 5-min test. The results of ambulatory activity were expressed as mean ± s.e.m.

### Perfusion

After FST (or under control condition) rats were anesthetized with ketamine (100 mg/kg, i.p., Indoketam® 1000, Virbac) and xilazine (20 mg/kg, i.m., Rompun®, Bayer) and perfused with a phosphate buffer solution (PBS: NaCl 0.13 M; NaH_2_PO_4_ 0.003 M; Na_2_HPO_4_ 0.007 M) and heparine (1 mL per liter of PBS; Inhepar®, Pisa) followed of 4% paraformaldehyde in PBS. Brains were removed, washed in PBS and preserved at 4°C in 30% sucrose and 0.1% timerosal in PBS. Afterwards, brain tissue was cut in a cryostat (−22°C, Microm HM 505 N) into coronal sections at 40 μm thick, which were preserved in a 30% sucrose and 0.1% timerosal in PBS at 4°C [26].

### Immunofluorescence

SERT-IR was examined on four animals from each group. Brain sections containing lateral septum (Bregma −0.24 mm) and dorsal raphe (Bregma −4.56) were identified following the Paxinos and Watson Atlas for rat brain [27]. Four adjacent sections of each area were taken for determination of SERT-IR. Brain sections were washed with PBS and nonspecific sites were blocked by incubation with solution A: a PBS solution containing 10% goat serum, 1% bovine serum albumine (BSA, Research Organics) and 0.3% Triton TX-100 (Sigma-Aldrich). Sections were placed in box and incubed for 1 h at room temperature under constant stir with the primary monoclonal antibody against the 1–85 a.a. N-terminal of the SERT, developed in mouse (Chemicon International) at a 1:500 dilution in solution A. Later, slides were incubated at 4°C constantly stirred overnight and later washed with 0.15% Triton TX-100 in PBS (Solution B). Brain sections were incubated at room temperature for 2 h with the secondary antibody (anti-IgG of mouse done in goat) marked with Oregon green 488 (Invitrogen®, Molecular Probes) dilution 1:100 in PBS with 5% goat serum and 0.3% Triton TX-100. Slides were washed with solution B, and mounted in a dark room on slides using antifade resin (Invitrogen®, Molecular Probes).

### Quantification of SERT immunoreactivity

A semiquantitative method was used to determine expression of SERT. Immunofluorescence was observed in a 40X oil immersion objective (SFluor, NA 1.3 Nikon) in an inverted microscope (Nikon Diaphot 300) equipped with an epifluorescence system (excitation: 480 ± 15 nm; dicroic mirrow: 505 nm; emission: 535 ± 20 nm) and coupled to a Xenon arc lamp (75 W). Images of SERT-IR were captured with a digital CCD camera (ORCA-ERC4742-95, Hamamatsu) and analyzed with the software MetaFluor version 6.1 (Universal Imaging Corporation). For each digitalized image a frequency histogram of fluorescence intensity was generated: in this histogram a threshold was established to eliminate non-specific fluorescence, pixels with fluorescence intensity above the threshold were considered specific for SERT-IR. The threshold (mean + 2.5 standard deviations) used in all preparations was established from 20 images of middle-aged animals brain structures. In these samples the referred threshold value effectively discarded background fluorescence. Once eliminated on specific fluorescence, the pixels with SER-IR were quantified and expressed as percentage relative to total pixels in the analyzed area (relative SERT-IR). This parameter was considered as an indicator of SERT expression. The parameters (illumination device: 488, slit 30 nm; time of exposure: 200 msec; gain: 100; and binding: 2) used to digitalize the images and the region of analysis (size: 670 × 512 pixels; area: 343040 pixels^2^) were constant across experimental groups and brain structures. A similar image analysis was used for other research groups to quantify proteins expression [28,29]. Quantification of SERT-IR was carried out bilaterally in the dorsal, intermediate and ventral portion of lateral septum, meanwhile in the raphe nuclei only dorsal raphe was analyzed.

### Statistical analysis

Influence of age in the FST and OFT behaviors was analyzed with a one-way ANOVA followed by a Tukey’s test when variance analysis attained statistical significance (p < 0.05). The relative SERT-IR area percentage was analyzed with a three-way ANOVA, considering the conditions of stress (control or FST), structure and age, followed by a Tukey’s test. When it was necessary, pairs of groups were compared with a Student *t* test.

## Results

### FST

**Figure 1:**
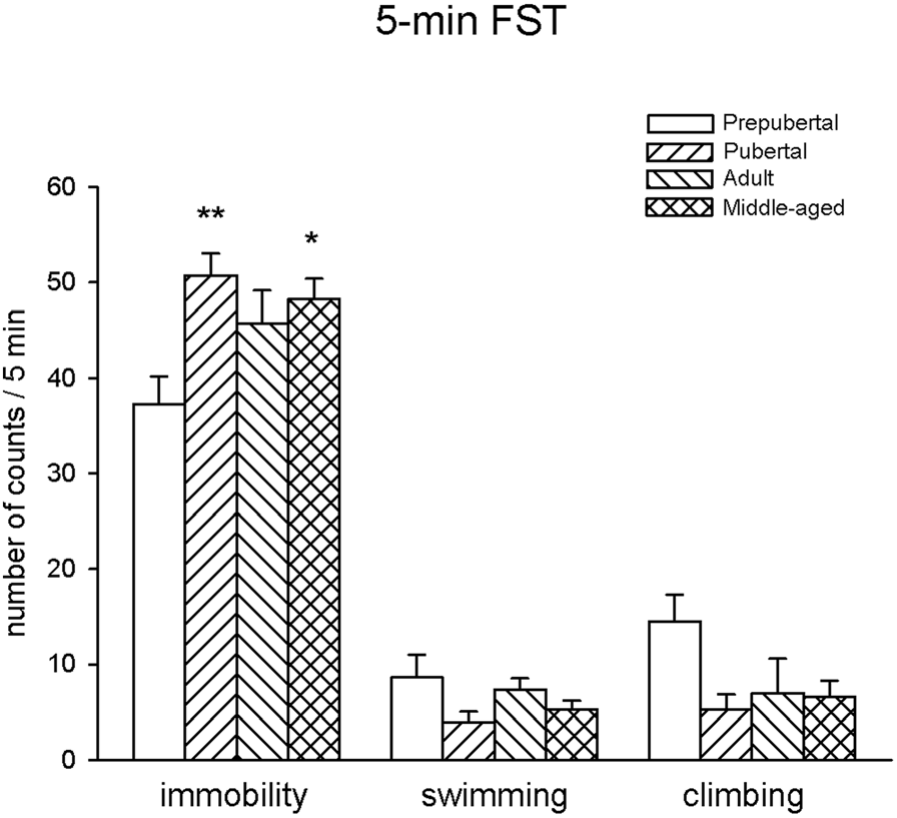
**FST behaviors of male rats in the 5-min session.** *p < 0.05, **p < 0.01 results of Tukey’s test vs. prepubertal rats.

In the test session statistical analysis showed differences in immobility behavior (F_3,28_ = 4.605, p = 0.01). Pubertal and middle-aged rats showed higher levels of immobility behavior compared to prepubertal rats. No significant differences were found in immobility of pubertal, adult and middle-aged rats. In addition, no significant variations in the expression of swimming (F_3,28_ = 1.65, p = 0.20) and climbing (F_3,28_ = 2.64, p = 0.06) was found (Figure 1).

### OFT

Statistical analysis showed differences in locomotor activity (Table 1, F_3,36_ = 22.948, p < 0.001). Prepubertal rats had lower levels of ambulatory activity in this test in comparison to the other groups. Ambulation was stabilized in pubertal and young adults and decreased in the middle-aged group, this response attained statistical significance respect to young adults.

**Table 1:** Ambulatory activity of male rats in a open field test

	**Number of counts/5 min**
Groups	
Prepubertal	786.60 ± 47.00
Pubertal	1640.90 ± 84.50^***^
Adult	1701.80 ± 108.30^***^
Middle-aged	1341.60 ± 96.99^***,#^

Results of the Turkey’s test: ***p < 0.001 vs. prepubertal rats, ^#^p < 0.05 vs. Adult.

### SERT-IR

**Figure 2:**
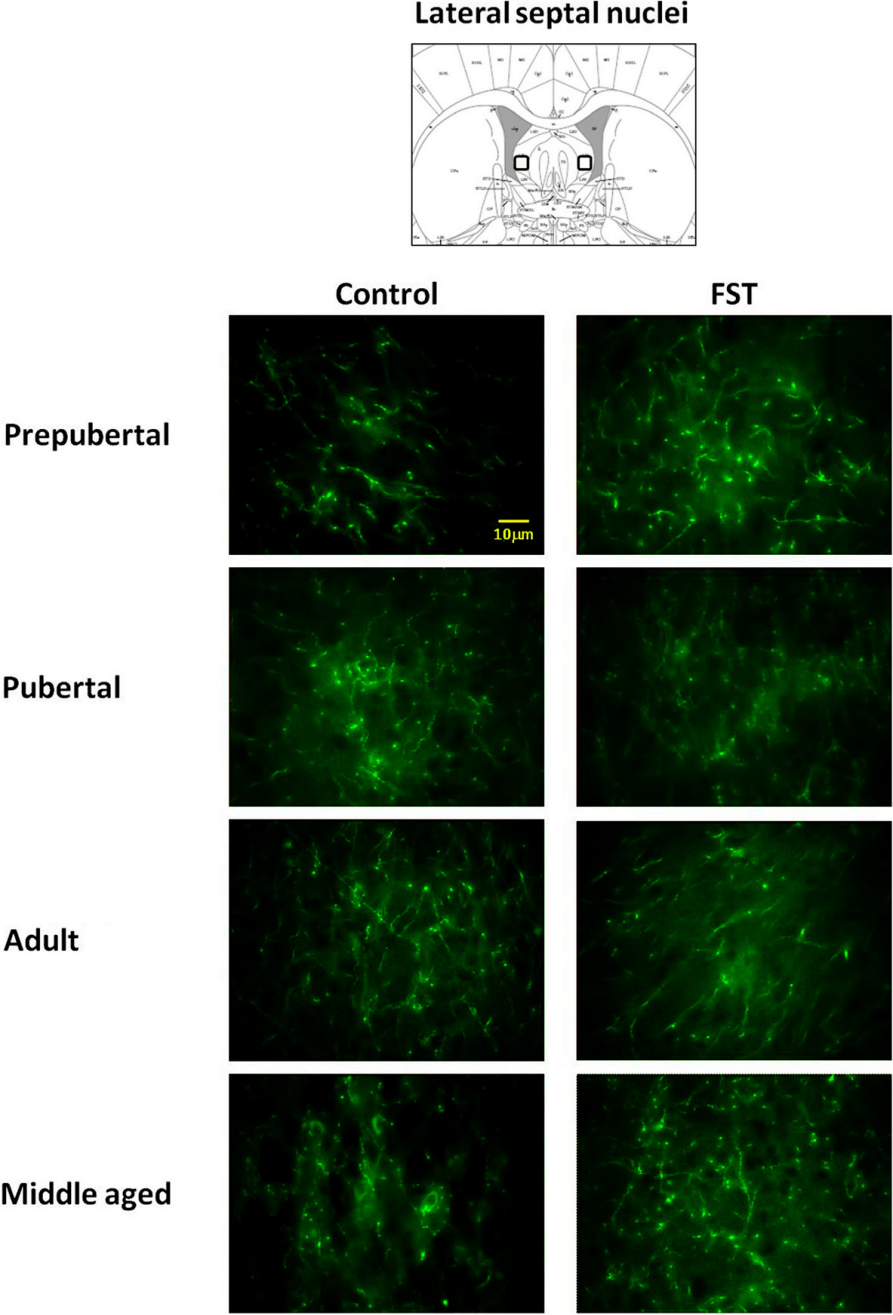
**Representative images of photomicrographs of SERT-IR in lateral septum of male rats.** Comparison between prepubertal, pubertal, adult and middle aged males subjected to FST vs. non-FST groups. The field of analysis is indicated in the upper slide (modified of Paxinos and Watson [27]).

**Figure 3:**
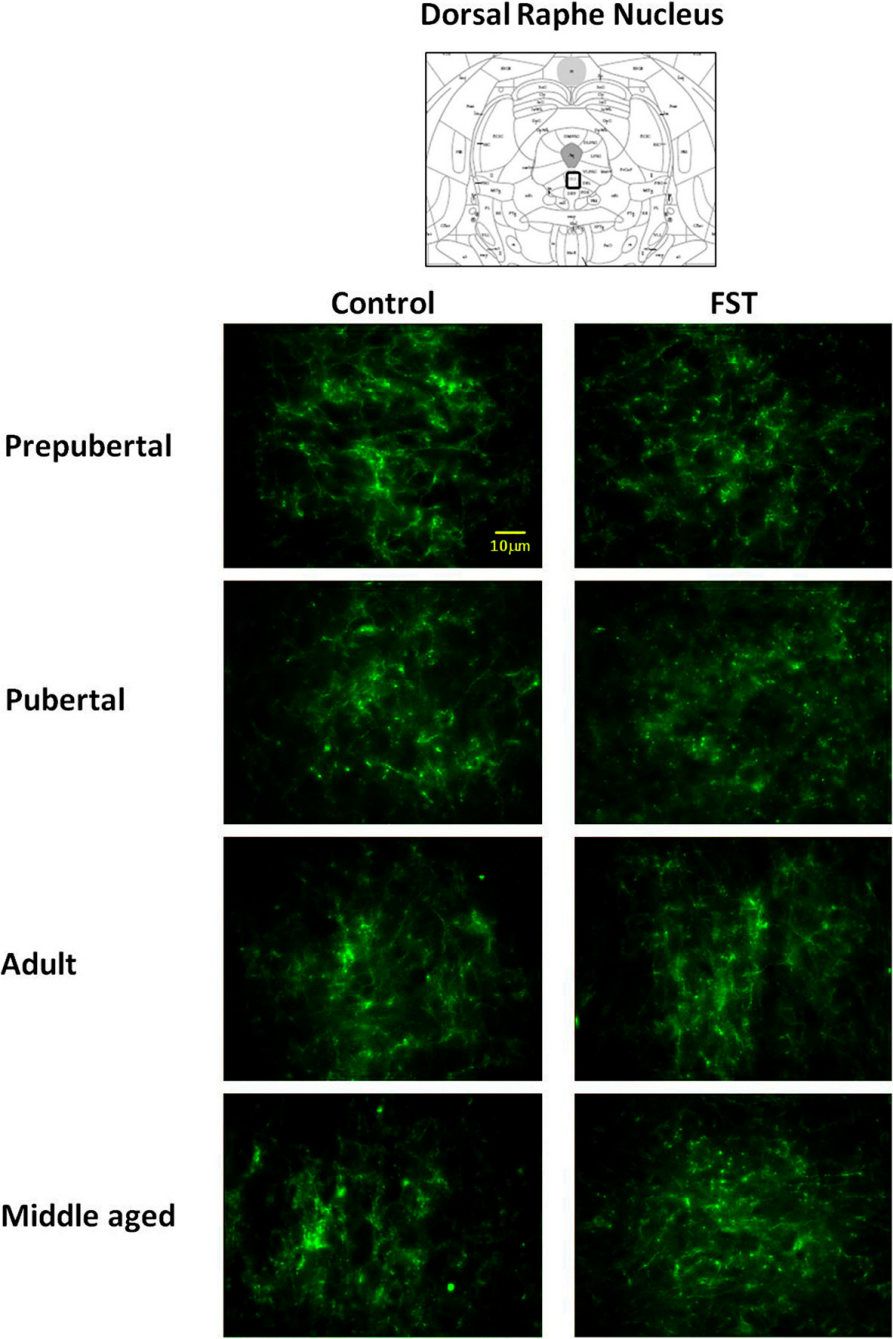
**Representative images of photomicrographs of SERT-IR in dorsal raphe of male rats.** Comparison between prepubertal, pubertal, adult and middle aged males subjected to FST vs. non-FST groups. The field of analysis is indicated in the upper slide (modified of Paxinos and Watson [27]).

Figures 2 and 3 show photomicrographs of SERT-IR in lateral septum and dorsal raphe, under control and FST conditions. Independently of age and stress condition, dorsal raphe exhibited fine and short punctuated fibers with varicosities (Figure 3). In contrast, lateral septum fibers were scarce but longer and wider than dorsal raphe’s (Figure 2). Analysis of relative SERT-IR quantified from the lateral septum and dorsal raphe of all animals showed a main effect of age (F_3,48_ = 4.284, p = 0.009) and brain structure (F_1,48_ = 25.56, p < 0.001) but not significant changes determined by stress (F_1,48_ = 0.39, p = 0.53), or the interaction between those factors (stress × age: F_3,48_ = 0.744, p = 0.058; stress × region: F_1, 48_ = 0.267, p = 0.608; age × region: F_3,48_ = 0.199, p = 0.331 and stress × age × region: F_3,48_ = 0.744, p = 0.531). Post-hoc analysis indicated that young adult rats showed larger relative SERT-IR than prepubertal (p = 0.036) and pubertal rats (p = 0.012). The dorsal raphe showed larger SERT-IR than lateral septum (p < 0.001).

**Figure 4:**
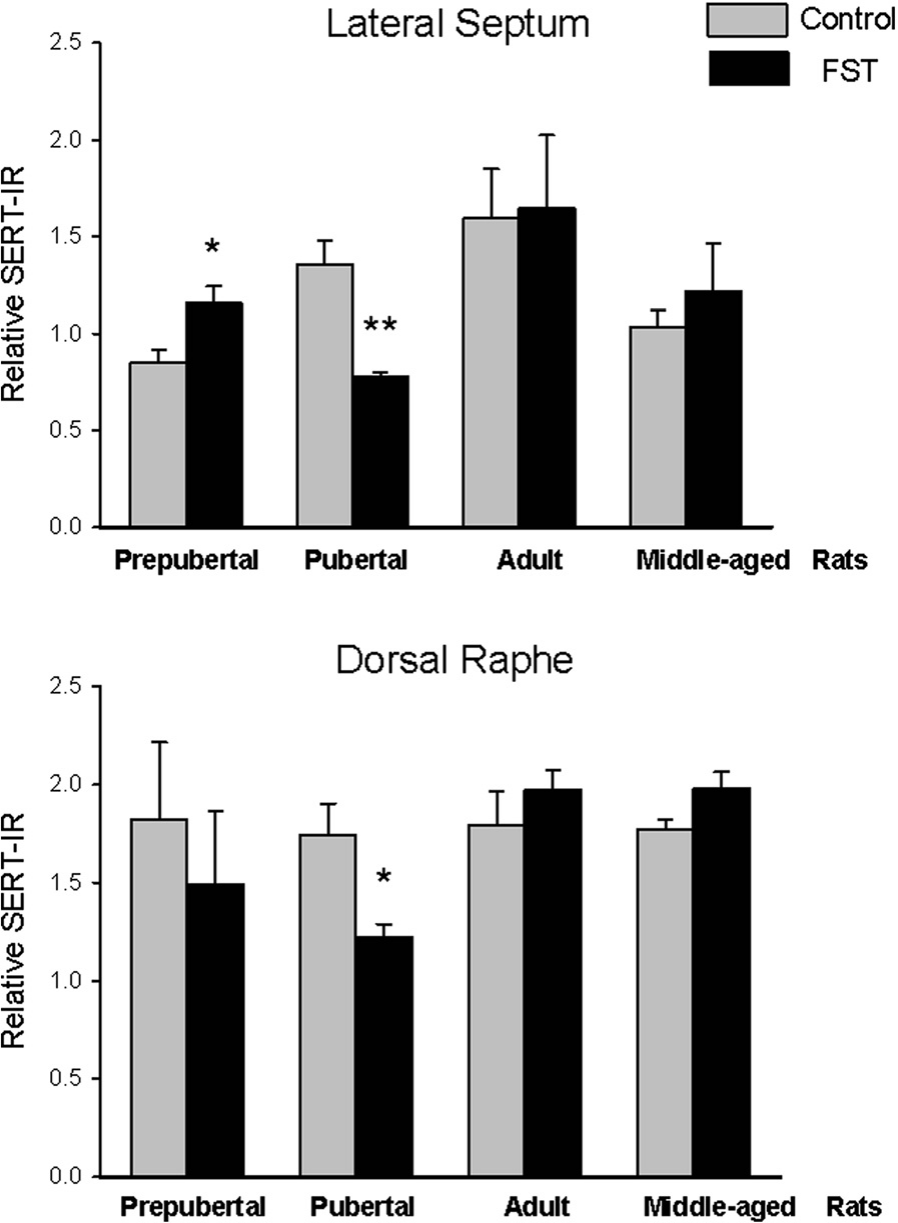
**Quantification of SERT-IR in lateral septum (upper graph) and dorsal raphe (lower graph).** Data are expressed as mean ± SEM. Results of *t* test: *p < 0.05, **p < 0.01 groups subjected to FST vs. non-FST (control).

Since the interaction stress × age tended to be statistically different (p = 0.058), we decided to evaluate relative SERT-IR differences determined by stress on each structure and for a single age. Differences by stress were observed on each structure, in lateral septum prepubertal subjects with FST showed a larger immunoreactive area than non-FST rats (t = −2.77, p = 0.032). In contrast, pubertal rats without FST exposure showed larger SERT-IR than the FST group in lateral septum (t = 4.60, p = 0.004) and dorsal raphe (t = 3.03, p = 0.02.) (Figure 4). No differences were found between relative SERT-IR of FST and non-FST groups of young and middle-aged adult rats.

## Discussion

Present results show that immobility behavior in the FST can be displayed by rats of different ages. Pubertal and middle-aged rats were more sensitive to the effects of forced swimming and showed increased levels of immobility respect to prepubertal and young adults. This study followed the methods described by Detke [13], where active behaviors (swimming and climbing) were measured, although younger animals displayed more active behaviors than the adults no significant age-related changes were observed.

Acute stress is used in animal models to induce behavioral, physiologic and neural changes relative to human depression [30]. The modified version of the FST [13] is a model that includes a pre-test session required to induce despair, reflected as an increase of immobility and a decrease of active behaviors in the test session [31,32]. The current study confirms the behavioral changes reported in adult rats and evidences a similar behavioral profile for prepubertal animals. The only study which evaluated the ontogeny of behaviors evaluated in the FST followed rats from 14 PN to 30 PN and reported that immobility emerges at day 21 PN and stabilizes beginning at day 26 PN [33]. To our knowledge, there are no studies examining immobility behavior from puberty; present results show that adult immobility levels are reached at puberty and stabilized from 40 days to 12 months since animals exhibited non significant variations (pubertal +36.24%, adult +22.81% and middle-aged rats +29.53%, respect to prepubertal). The influence of body weight could be discharged since behavior of adult males did not differ from pubertal ones, despite that the former group is almost 200% heavier than the later. Another possible explanation for the differences on immobility could be related with an age-dependent reduction of locomotor activity [34]; however, present results in the OFT contradict this idea, given that animals with lower ambulation (i.e. prepubertal ones) showed reduced immobility in the FST; in turn, middle-aged and young adult rats expressed similar immobility, but the former group showed reduced ambulation*.*

It can be argued that the developmental related changes in serotonergic system influence directly the observed changes on immobility in rats of different ages. Gallineau and colleagues showed that SERT density measured in dorsal raphe and parietal cortex peaks and declines prior to PN 20, these changes were suggested to be secondary to a peak in extracellular 5-HT during brain development [35]. To our knowledge, there are no studies examining lifespan SERT-IR in rodents, thus present results showed a pubertal and adulthood increase followed by a moderate decline in middle age. In this line, a radiobinding study in non-human primates have shown that aging is associated with a SERT specific binding decrease, which was related to the hyperactivity of Hypothalamus-Pituitary-Adrenal axis [4].

Present results suggest that FST reduces SERT-IR in lateral septum of pubertal rats, which could be related to the higher expression of immobility observed in these animals. A 5-HT decrease in lateral septum after pretest was related to behavioral despair [19], which is prevented by fluoxetine [18]. Changes in the lateral septum SERT-IR could be secondary to 5-HT concentration or changes in the transcription [17]. Previous studies of Lucki et al. suggested that changes FST induces CRH release on 5-HT neurons of dorsal raphe; this peptide acting through CRF2 receptors reduces discharge rate of dorsal raphe neurons, which send projections to lateral septum; specifically, the dorsal dorsal raphe has a functional relationship with lateral septum, and modulates 5-HT levels in lateral septum. This neurochemical change could be directly related to immobility [17]. Studies have revealed an age- and androgen-dependent regulation of CRF2 binding in rat intermediate lateral septum, showing an increased functionality from puberty to adulthood [36]. Based on this idea, present results show that prepubertal animals exhibit less depressive-like behaviors and more SERT expression than pubertal animals. According to Blakely’s hypothesis [37], the regulation of the transporter protein in the presynaptic membrane is more dependent on the concentration of serotonin in the synapse than driven by gene expression according to the “use it or lose it”, so our results could suggest that 5-HT levels are higher in prepubertal animals, leading to a higher expression of SERT, and thus less depressive behaviors in response to FST. Further studies are needed to confirm this.

According to the model proposed by Sheehan [38], lateral septum is a retrieval structure implicated on stress coping through connections with amygdala and hippocampus; further studies on the effect of FST in SERT-IR of these structures are needed to obtain a more integrative model for depressive-like behavior.

The lack of effect of FST in adult’s SERT-IR could be explained by the fact that this stressor does not affect adult’s SERT expression on this structures, previous studies had shown changes on SERT mRNA following an acute stress [10], although some reports showed differences in mRNA and protein expression [39], suggesting that these changes could take more or less time to be seen, so a time-course of SERT-IR should be made to detect them.

## Conclusions

Pubertal rats were more susceptible to the effects of stress by forced swimming test on structures involved on the expression of behavioral despair. Present results increase the evidence of age-dependent susceptibility to stress, which may model clinical characteristics and treatment response of depression throughout life.

## Abbreviations

FST: Forced swimming test; OFT: Open field test; SERT: Serotonin transporter; SERT-IR: SERT immunoreactivity; PN: Postnatal day; SSRIs: Selective serotonin reuptake inhibitors.

## Competing interests

The authors declare that they have no competing interests.

## Authors’ contributions

REU and LMM designed the study and drafted the manuscript, ADV and JHP carried out the behavioral tests and immunofluorescence technique, MLO supervised the immunoassays and the elaboration of the figures. All authors reviewed and approved the final manuscript.
